# Color-encoded single-shot computer-generated Moiré profilometry

**DOI:** 10.1038/s41598-021-90522-x

**Published:** 2021-05-26

**Authors:** Hechen Zhang, Yiping Cao, Chengmeng Li, Lu Wang, Hongmei Li, Cai Xu, Yingying Wan

**Affiliations:** 1 Department of Optical Electronics, Sichuan University, Chengdu, 610064 China; 2 College of Physics and Engineering, Chengdu Normal University, Chengdu, 611130 China

**Keywords:** Optics and photonics, Optical techniques, Engineering, Electrical and electronic engineering

## Abstract

A color-encoded single-shot computer-generated Moiré profilometry (CSCGMP) is proposed. Two sinusoidal gratings with a *π* phase difference are encoded in red and blue channels respectively to combine a composite color grating. While this composite color grating is projected onto the measured object, the corresponding color deformed pattern can be captured. So two deformed patterns with a *π* phase difference are separated from its red and blue components respectively. After normalization and subtraction, the AC component of both separated deformed patterns can be extracted. If this AC component respectively multiplied by the two AC components of fringe patterns of reference plane with a *π*/2 phase difference prepared and saved on the computer in advance, two computer-generated Moiré fringes just respectively standing for sine and cosine of phase which is modulated by the height of the object relative to the reference plane are figured out. So the 3D shape of the measured object can be reconstructed with normal computer-generated Moiré profilometry. Both simulation and experimental results show the feasibility and validity of the proposed method. It has potential in real-time 3D measurement due to its single-shot feature.

## Introduction

Nowadays, three-dimensional (3D) measurement^1–6^ is applied widely, such as reverse engineering^7,8^, three dimensional print^9^, medical treatment^10^ and precision instrument testing^11^. 3D measurements can be classified as touchable measurements and untouchable measurements. Cause untouchable measurements have the advantages of no damage to objects, portable setup, and fast measuring speed^12–15^, they are getting a lot of attention.

Among these untouchable measurements, Fourier-transform profilometry (FTP)^16^ proposed by Takeda is proper to achieve fast measurement. However, because the wrapped phase is obtained by filtering out the either of the two spectra on the carrier frequency which contains the 3D information of the measured object, FTP is hard to obtain higher accuracy. To improve this method, some methods were proposed by some researchers^17–20^. Furthermore, phase measuring profilometry (PMP)^21^ proposed by Srinnivasan is proper to achieve higher accuracy measurement by using several shots. To actualize real-time measuring and reserve the superiority of PMP, a single-shot color phase-shifting technique (CPST)^22,23^ was proposed. In this method, three phase-shifting sinusoidal gratings are encoded in red, green, and blue channels to combine the projected color grating. And then a composite grating phase measuring profilometry (CGPMP) was proposed^24^. In this method, three phase-shifting sinusoidal gratings are modulated by three other orthogonal carrier gratings with different frequencies to combine the projected composite grating. Though this method can realize real-time measurement, the dynamic range is limited because six or more gratings share at most 256 Gy levels. Although Moiré profilometry (MP) was proposed by Takasaki early in 1970^25^, its development has been severely challenged, because it is based on analyzing the Moiré fringes caused by two overlapped gratings and its optical setup is more complex than that of FTP or PMP. Recently, along with the development of computer technology, a computer-generated Moiré profilometry (CGMP)^26^ was proposed. It just captures only one deformed pattern rather than directly generates and captures Moiré fringe like MP, and the Moiré fringes are generated just by the computer. It has the advantages of achieving real-time measurement as FTP and realizing relatively high precision measurement as PMP. But when the zero-order spectrum component is overlapped by the first-order spectrum component in the captured deformed pattern, the measuring accuracy of CGMP may be limited. To achieve higher accuracy, high precision computer-generated Moiré profilometry (HCGMP)^27^ was proposed. However, the feature of single-shot projection may be challenged to some extent because two deformed patterns are needed to be captured in the measuring.

To solve this problem, a color-encoded single-shot computer-generated Moiré profilometry (CSCGMP) is proposed. In this method, a composite color grating is composed of a sinusoidal grating encoded in the red channel and a sinusoidal grating with a *π* phase difference encoded in the blue channel. While this composite color grating is projected onto the measured object, only a single-shot color deformed pattern is needed to be captured. Then, the AC component of the color deformed pattern is extracted by color separating and normalization rather than spectrum filtering. So the 3D measuring accuracy can be improved.

## Principle

**Figure 1:**
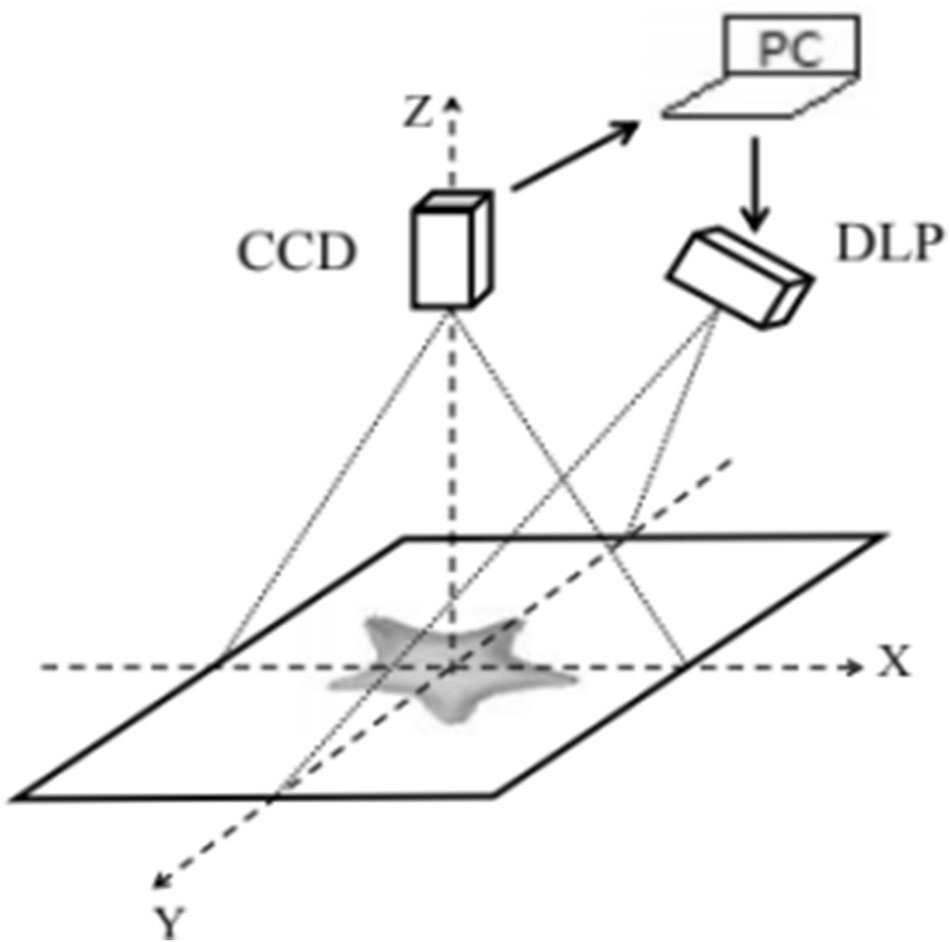
The setup of CSCGMP.

The setup of CSCGMP is the same with that of CGMP as shown in Fig. 1. It is composed of a CCD camera, a projector (DLP), and a computer (PC).

Before measuring, preparation work should be carried out. Firstly, four gray-scale sinusoidal gratings with a *π*/2 phase difference in the sequence are projected onto the reference plane. The corresponding four gray-scale sinusoidal patterns modulated by the reference plane are captured as follows:

1 $$I_{0} (x,y) = R(x,y)\{ A + B\cos [2\pi fx + \varphi_{0} (x,y)]\}$$

2 $$I_{{1}} (x,y) = R(x,y)\{ A + B\cos [2\pi fx + \varphi_{0} (x,y) + \pi /2]\}$$

3 $$I_{{2}} (x,y) = R(x,y)\{ A + B\cos [2\pi fx + \varphi_{0} (x,y) + \pi ]\}$$

4 $$I_{{3}} (x,y) = R(x,y)\{ A + B\cos [2\pi fx + \varphi_{0} (x,y) + 3\pi /2]\}$$

where *R*(*x*, *y*) stands for the reflectance of the reference plane, *A* stands for the background light intensity, *B* reflects the fringe contrast, and *ϕ*_0_(*x*, *y*) presents the phase distribution modulated by the reference plane.

To get rid of the background light intensity, *I*_0_(*x*, *y*) should minus *I*_2_(*x*, *y*), and *I*_1_(*x*, *y*) should minus *I*_3_(*x*, *y*). These expressions are shown as follows:

5 $$I_{\cos } (x,y) = 0.5(I_{0} - I_{2} ) = BR(x,y)\cos [2\pi fx + \varphi_{0} (x,y)]$$

6 $$I_{\sin } (x,y) = 0.5(I_{1} - I_{3} ) = BR(x,y)\cos [2\pi fx + \varphi_{0} (x,y) + \pi /2]$$

where *I*_cos_(*x*, *y*) and *I*_sin_(*x*, *y*) are just the AC components of *I*_0_(*x*, *y*) and *I*_1_(*x*, *y*) respectively. They are stored in the computer in advance.

**Figure 2:**
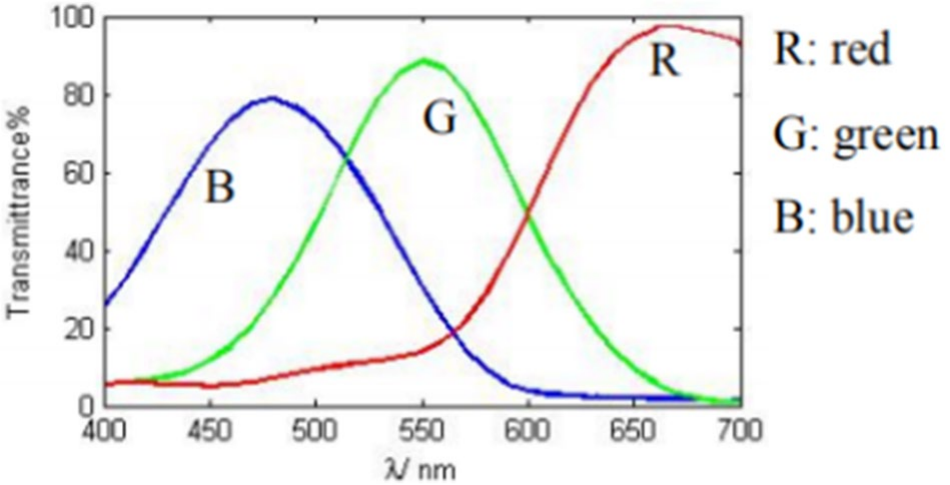
Spectral coupling curve of CCD.

Then, a composite color grating in which two sinusoidal gratings with a *π* phase difference each other are encoded in the red channel and the blue channel respectively is designed and also stored on the computer in advance. The reason why the red channel and the blue channel are chosen is much dependent on the spectral coupling curve of the CCD camera as shown in Fig. 2. It can be seen the color coupling between the blue channel and the red channel is the smallest.

In the measuring work, while the designed composite color grating is projected onto the measured object, the corresponding color deformed pattern modulated by both the measured object and the reference plane can be captured. The two deformed patterns separated from the red channel and blue channel of the captured color deformed pattern can be expressed below:

7 $$I_{r} (x,y) = R_{r} (x,y)\{ A + B\cos [2\pi fx + \varphi (x,y)]\}$$

8 $$I_{b} (x,y) = R_{b} (x,y)\{ A + B\cos [2\pi fx + \varphi (x,y) + \pi ]\}$$

where *R*_r_(*x*, *y*) presents the red light reflectance of the measured object surface, and *R*_b_(*x*, *y*) presents the blue light reflectance of the object surface, *A* stands for the background light intensity, *B* reflects the fringe contrast, and *ϕ*(*x*, *y*) stands for the phase distribution modulated by both the measured object and the reference plane.

It can be seen from Fig. 2, the red light responsiveness and blue light responsiveness of the CCD are different, so *AR*_*r*_(*x*, *y*) and *AR*_*b*_(*x*, *y*), and *BR*_*r*_(*x*, *y*) and *BR*_*b*_(*x*, *y*) are different respectively. *BR*_*r*_(*x*, *y*) and *BR*_*b*_(*x*, *y*) called fringe modulations are filtered out respectively as followings:

9 $$I_{m\_r} (x,y) = abs(2*FFT^{ - 1} \{ Filter(f_{x0} ,f_{y0} )*FFT[I_{r} (x,y)]\} ) = BR_{r} (x,y)$$

10 $$I_{m\_b} (x,y) = abs(2*FFT^{ - 1} \{ Filter(f_{x0} ,f_{y0} )*FFT[I_{b} (x,y)]\} ) = BR_{b} (x,y)$$

Considering the modulations in shadow areas are close to zero, a binary mask is generated according to the threshold of modulation based on Otsu^28^ to avoid errors caused by shadow.

After using the mask, two separated deformed patterns are normalized as follows:

11 $$I_{normal\_r} (x,y) = I_{r} (x,y)/I_{m\_r} (x,y) = A/B + \cos [2\pi fx + \varphi (x,y)]$$

12 $$I_{normal\_b} (x,y) = I_{b} (x,y)/I_{m\_b} (x,y) = A/B + \cos [2\pi fx + \varphi (x,y) + \pi ]$$

It can be seen from Eqs. (11 and 12), there is a *π* phase difference between $I_{normal\_r} (x,y)$ and $I_{normal\_b} (x,y)$, so the AC component of the two separated deformed patterns could be extracted:

13 $$I_{AC} (x,y) =0.5[ I_{normal\_r} (x,y)-I_{normal\_b} (x,y)] = \cos [2\pi fx + \varphi (x,y) ]
$$

Then, by multiplying this AC component with the above two AC components prepared in advance, two equivalent transmittances of the overlapping gratings generated by the computer can be respectively expressed as follows:

14 $$\begin{gathered} I_{t1} (x,y) = I_{AC} (x,y)I_{\cos } (x,y) \hfill \\ = 2BR(x,y)\{ \cos [4\pi fx + \varphi (x,y) + \varphi_{0} (x,y)] + \cos [\varphi (x,y) - \varphi_{0} (x,y)]\} \hfill \\ \end{gathered}$$

15 $$\begin{gathered} I_{t2} (x,y) = I_{AC} (x,y)I_{\sin } (x,y) \hfill \\ =- 2BR(x,y)\{ \sin [4\pi fx + \varphi (x,y) + \varphi_{0} (x,y)] - \sin [\varphi (x,y) - \varphi_{0} (x,y)]\} \hfill \\ \end{gathered}$$

The two computer-generated Moiré fringes are just the DC components of these two equivalent transmittances. A filter is designed to filter out the DC components of these two equivalent transmittances. The computer-generated Moiré fringes are shown as:

16 $$I_{Moire0} (x,y) = FFT^{ - 1} \{ Filter(f_{x1} ,f_{y1} )*FFT[I_{t1} (x,y)]\} = 2BR(x,y)\cos [\varphi (x,y) - \varphi_{0} (x,y)]$$

17 $$I_{Moire90} (x,y) = FFT^{ - 1} \{ Filter(f_{x1} ,f_{y1} )*FFT[I_{t2} (x,y)]\} = 2BR(x,y)\sin [\varphi (x,y) - \varphi_{0} (x,y)]$$

Finally, the phase information of the height of the measured object relative to the reference plane can be obtained:

18 $$\Delta \varphi_{1} (x,y) = \varphi (x,y) - \varphi_{0} (x,y) = \arctan (I_{Moire90} /I_{Moire0} )$$

Because of the arctangent operation, the phase Δ*ϕ*_1_(*x*, *y*) is wrapped in (− *π*, *π*]. The phase unwrapping method is used to calculate the unwrapped phase Δ*ϕ*_2_(*x*, *y*) from the wrapped phase^29^. After the height-to-phase mapping^30^, the height of the measured object can be obtained:

19 $$1/h(x,y) = a(x,y) + b_{1} (x,y)/\Delta \varphi_{2} (x,y) + b_{2} (x,y)\varphi_{0} (x,y)/\Delta \varphi_{2} (x,y) + c(x,y)/\Delta \varphi_{2}^{2} (x,y)$$

where *a*(*x*, *y*), *b*_1_*a*(*x*, *y*), *b*_1_(*x*, *y*), *b*_2_(*x*, *y*), and *c*(*x*, *y*) are the system parameters. They can be obtained by calibration.

**Figure 3:**
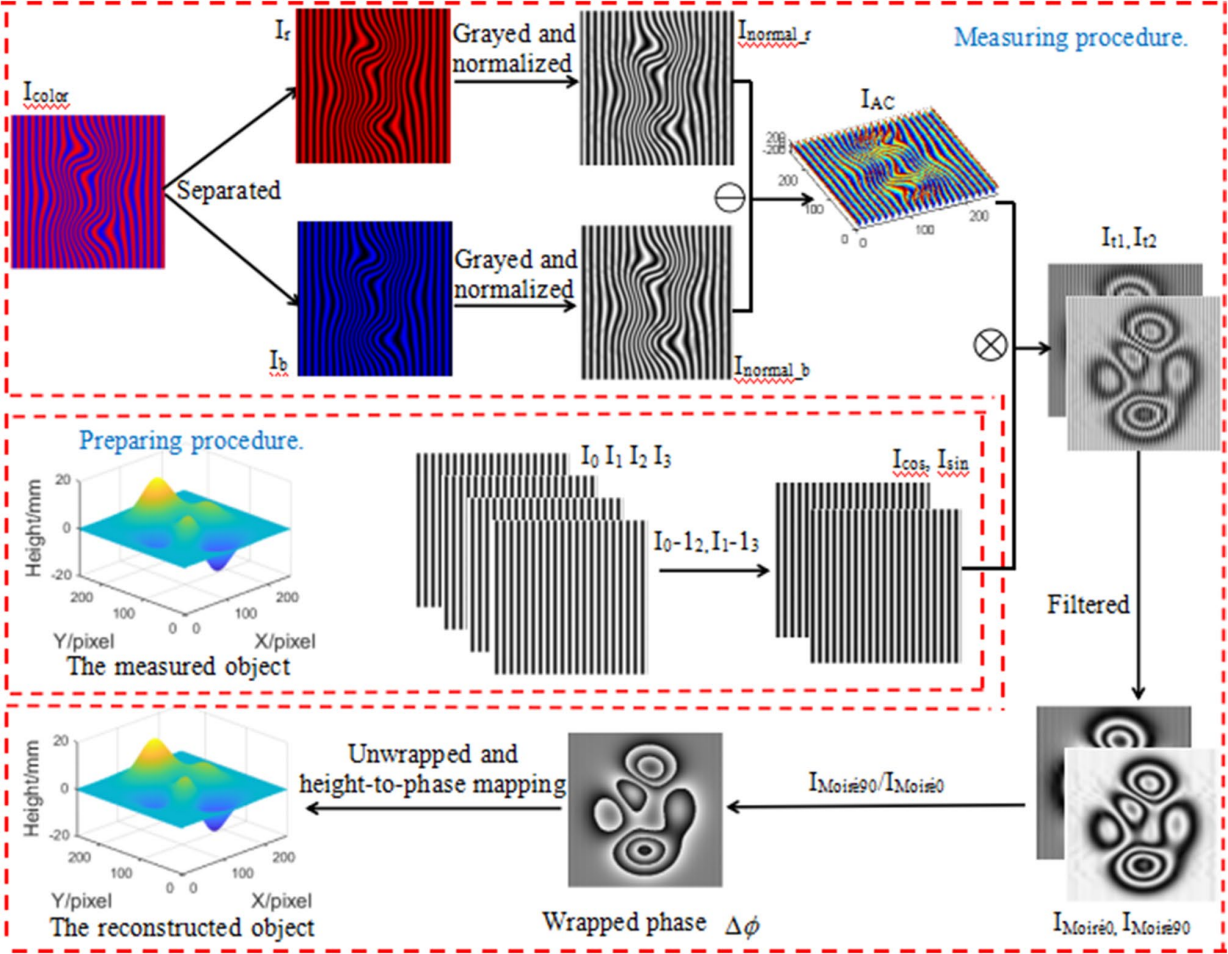
The principle flow chart of CSCGMP.

In summary, the flow chart of CSCGMP is as shown in Fig. 3.

## Simulation

**Figure 4:**
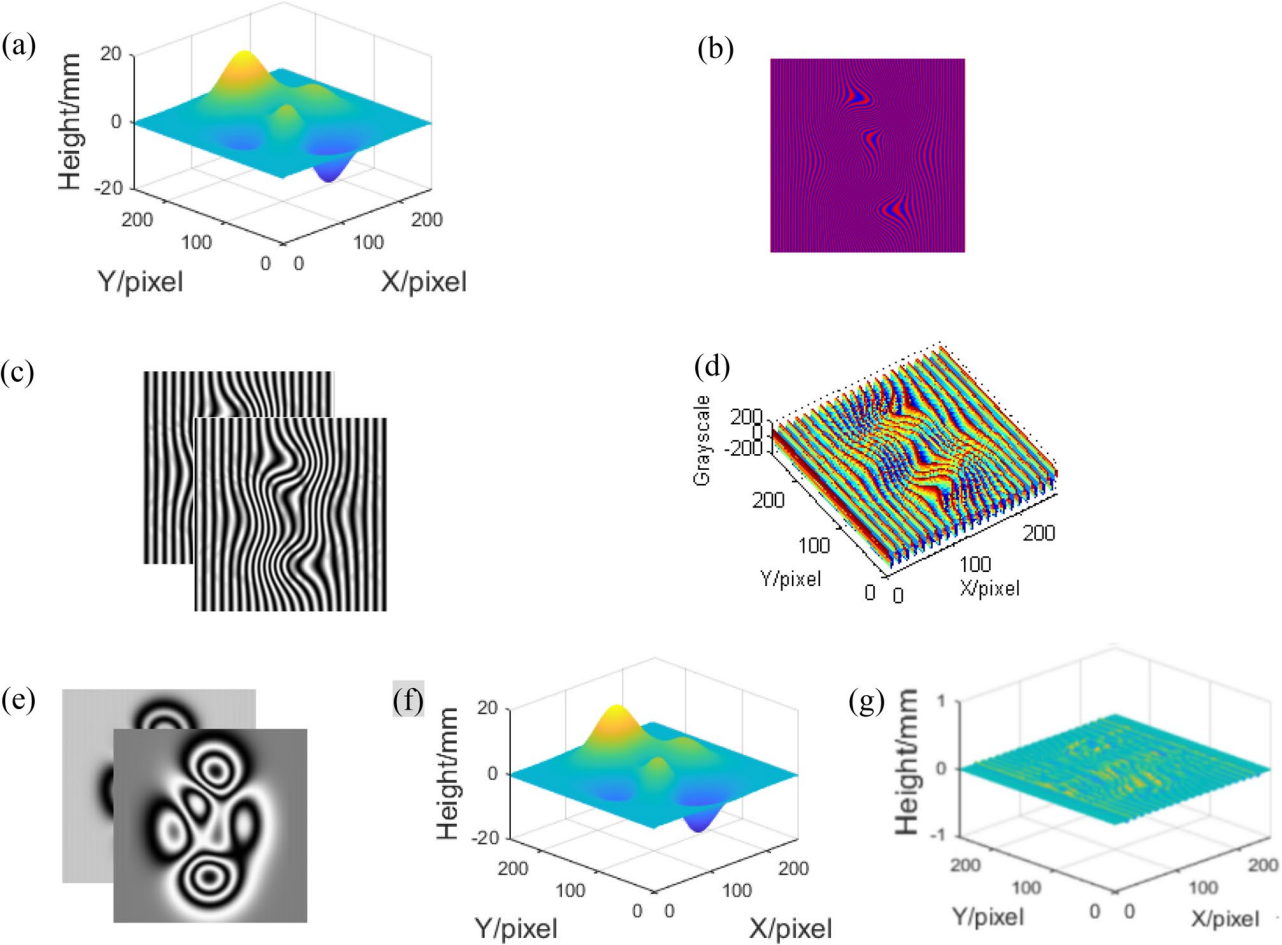
Simulation of color-encoded single-shot computer-generated Moiré profilometry: (**a**) the measured peaks; (**b**) the color deformed pattern of the peaks; (**c**) the red and blue components after normalization of the peaks; (**d**) the AC component I_AC_ of the peaks; (**e**) the Moiré0 and Moiré90 fringes of the peaks; (**f**) the reconstructed peaks; (**g**) the error with CSCGMP.

In order to verify the feasibility of CSCGMP, lots of simulations have been done. Here, a peaks function as shown in Fig. 4a is designed as the measured object. Before measuring, two AC components of the fringe patterns of the reference plane with a *π*/2 phase difference are prepared in advance. Figure 4b shows the corresponding color deformed pattern whose phase is modulated by the height of the peaks relative to the reference plane, and Fig. 4c shows the red component and the blue component of the color deformed pattern after normalization. Figure 4d is the AC component of the red deformed pattern after normalization in Fig. 4c. Figure 4e shows the computer-generated Moiré fringes Moiré0 and Moiré90. Figure 4f shows the reconstructed object. It can be seen from Fig. 4f that the reconstructed object is rebuilt well by CSCGMP. What’s more, in order to verify CSCGMP’s accuracy, the error distribution of the reconstructed object is measured as shown in Fig. 4g. It is recognized that the error with CSCGMP is small enough, the maximum error is 0.0652 mm, the minimum value is − 0.0738 mm, and the root of mean square (RMS) is 0.0892 mm.

## Experimental results and analysis

In order to prove the validity of CSCGMP, large numbers of 3D measurement experiments are carried out. The setup is as shown in Fig. 1, the type of the CCD is DFM 72AUC02, and the type of the DLP is ViewSonic PLED-W200.

**Figure 5:**
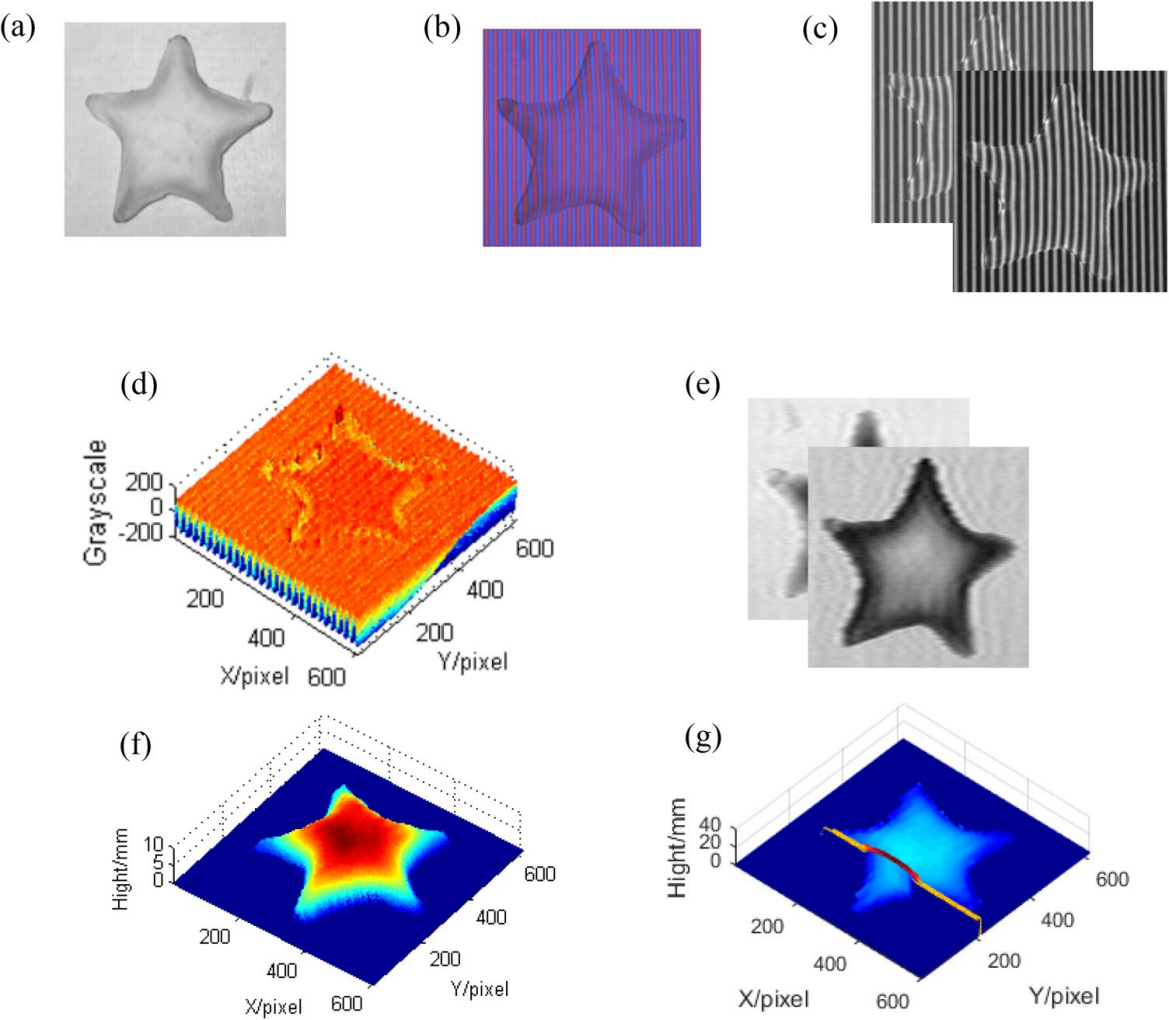
The reconstructed results of the star: (**a**) the measured star; (**b**) the color deformed pattern; (**c**) the normalized red and blue components; (**d**) the AC component of the red normalized deformed pattern; (**e**) Moiré0 and Moiré90 fringes; (**f**) the reconstructed star with normalization and binary mask; (**g**) the reconstructed star without normalization and binary mask.

In preparing procedure, the four gray-scale sinusoidal gratings are projected onto the reference plane sequentially, and the corresponding four gray-scale sinusoidal patterns are captured by the CCD camera. The AC components of fringe patterns of the reference plane with a *π*/2 phase difference can be obtained according to Eqs. (5 and 6) and are saved on the computer. Then, in the measuring procedure, a star model as shown in Fig. 5a is put on the reference plane, and the designed composite color grating is projected onto the star. The corresponding color deformed pattern is captured by the CCD camera as shown in Fig. 5b, and it is also saved on the computer. After the red component and the blue component are separated from the color deformed pattern, the normalized deformed patterns are as shown in Fig. 5c. The AC component of the red normalized deformed pattern can be get as shown in Fig. 5d. Two computer-generated Moiré fringes are as shown in Fig. 5e. Finally, the 3D shape of the measured star model can be reconstructed, and the reconstructed result is shown in Fig. 5f. It can be seen from Fig. 5f that CSCGMP can reconstruct the object well with normalization and binary mask. Figure 5g shows the reconstructed star without normalization and binary mask. From comparing Fig. 5f and Fig. 5g, it can be seen that the error can be eliminated as much as possible by introducing normalization and binary mask. Because only one color-encoded composite grating is needed to be projected onto the measured object and only one corresponding composite color deformed pattern is needed to be captured, it still reserves the single-shot feature and plays a potential application in real-time 3D measurement.

**Figure 6:**
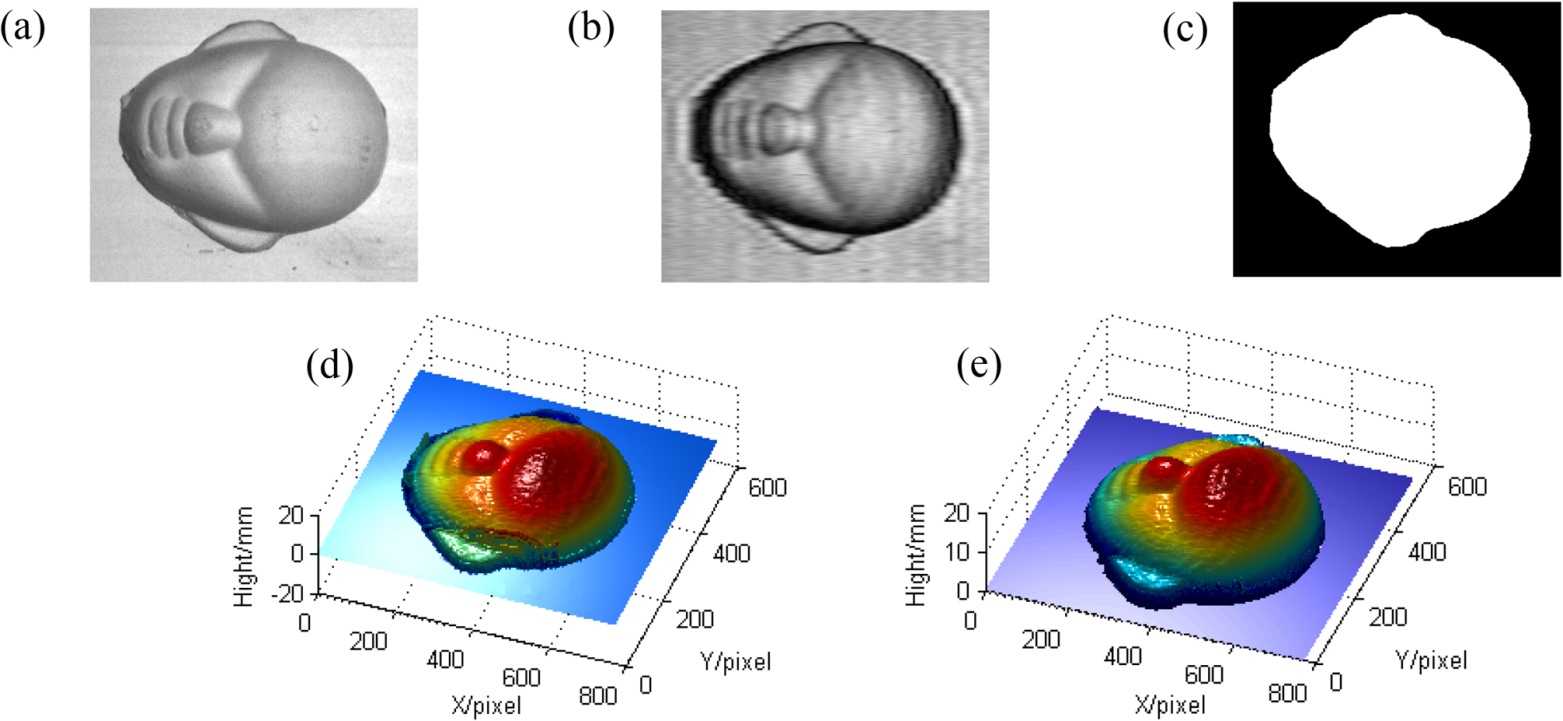
The comparison of reconstructed face models before and after using the mask: (**a**) the measured face; (**b**) the modulation; (**c**) the mask; (**d**) the reconstructed face before using the mask; (**e**) the reconstructed face after using the mask.

In order to reveal that the errors caused by shadow can be efficiently avoided by using the mask in CSCGMP, a face model is measured as shown in Fig. 6.

Figure 6a shows the measured face model. Figure 6b shows the modulation which is filtered from the red component of the captured color deformed pattern, and Fig. 6c shows the designed binary mask based on the Otsu method. Figure 6d, e are the reconstructed results before and after using the mask. It can be seen that the reconstructed face model after using the mask is more reliable and more accurate.

**Figure 7:**
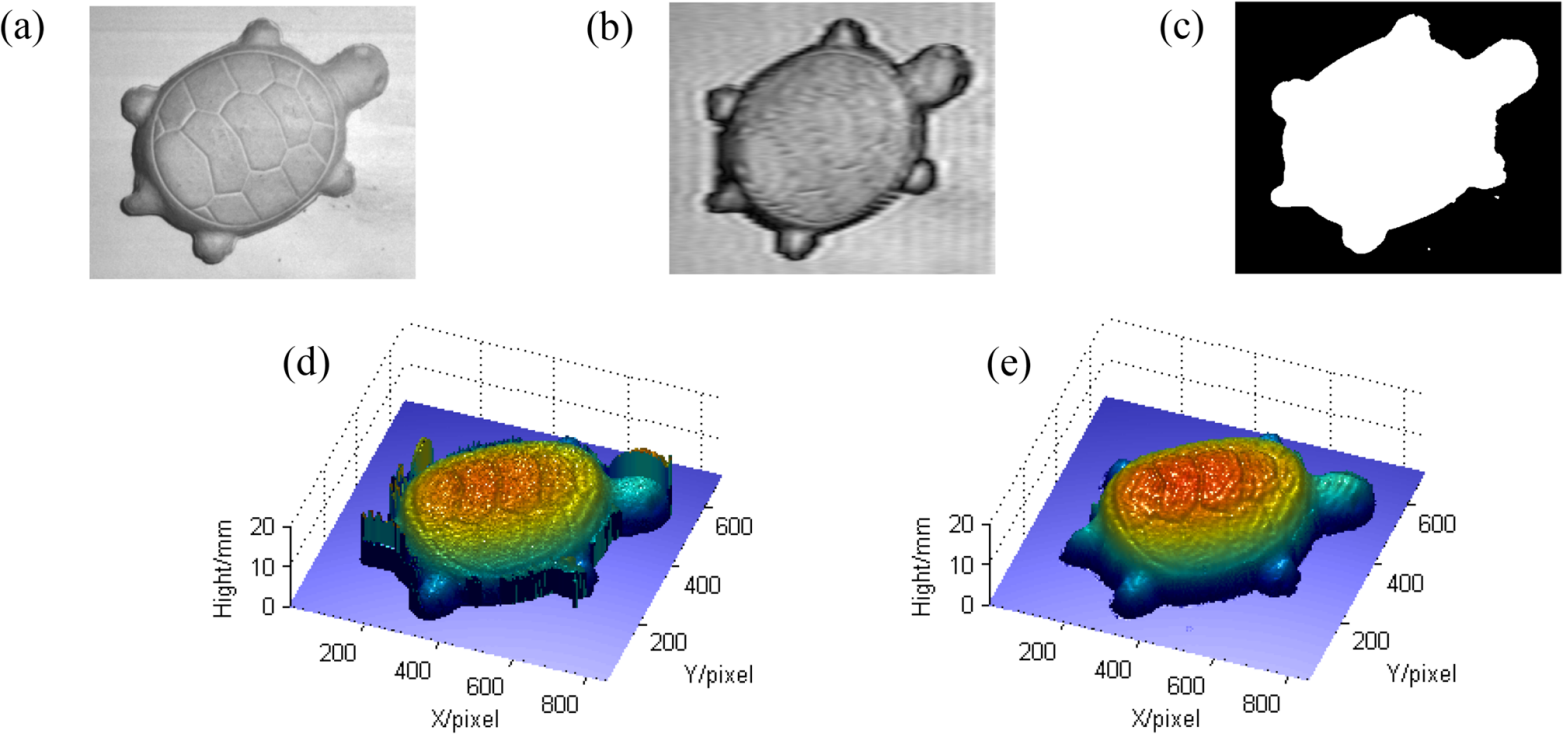
The comparison of reconstructed turtle models before and after using the mask: (**a**) the measured turtle; (**b**) the modulation; (**c**) the mask; (**d**) the reconstructed turtle before using the mask; (**e**) the reconstructed turtle after using the mask.

In addition, a turtle model with more detailed textures is also measured as shown in Fig. 7. Figure 7a shows the measured turtle model. Figure 7b shows the modulation which is filtered from the red component of the captured color deformed pattern, and Fig. 7c shows the binary mask. Figure 7d, e are the reconstructed results before and after using the mask. It can be seen that the reconstructed turtle after using the mask is more reliable and shows more detailed texture information. It further reveals that the proposed method is more applicable and more realistic.

In order to verify the proposed CSCGMP can be applied into real-time measurement, a lot of dynamic objects have been measured by CSCGMP.

**Figure 8:**
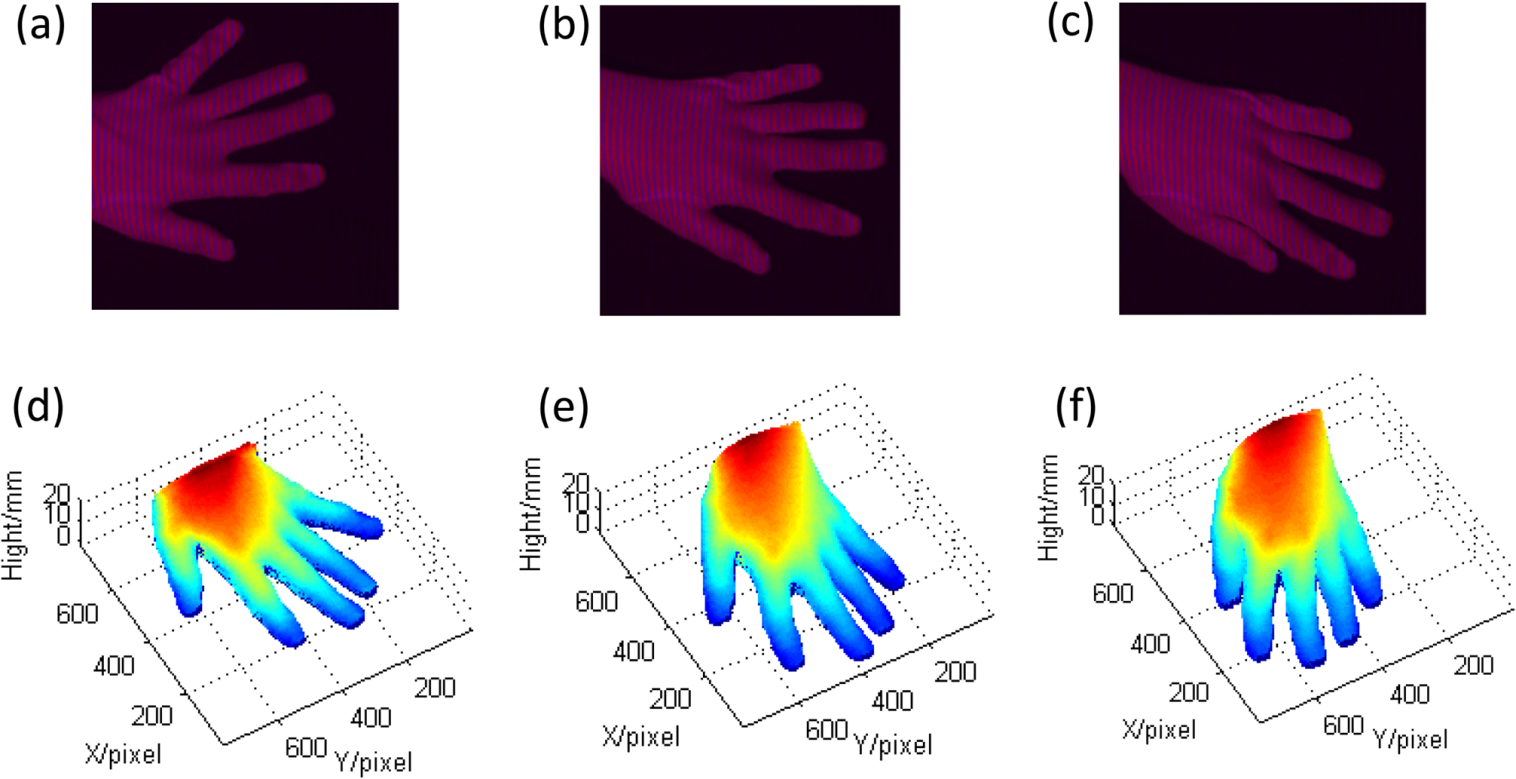
The measuring results of the dynamic hand at three random states. (**a**)–(**c**) The captured color deformed patterns at state1, state2, and state3, and they are integrated in Supplementary Video 1 (Supplementary Video 1, MP4, 1.16 MB); (**d**)–(**f**) the reconstructed hands at state1, state2, and state3, and they are integrated in Supplementary Video 2 (Supplementary Video 2, MP4, 346 KB).

Figure 8 shows the measuring results of a moving hand which is one of the authors by CSCGMP. The 3D shapes of the real-time dynamic hand are obtained at 42 fps, and the execution time is 0.0238 s/frame. Three different dynamic states of the moving hand are shown in Fig. 8.

In Fig. 8, it can be seen that the dynamic hand is reconstructed well by CSCGMP. Figure 8a–c show the captured color deformed patterns at three random times in real-time measurement, and Fig. 8d–f show the reconstructed hands at corresponding times.

**Figure 9:**
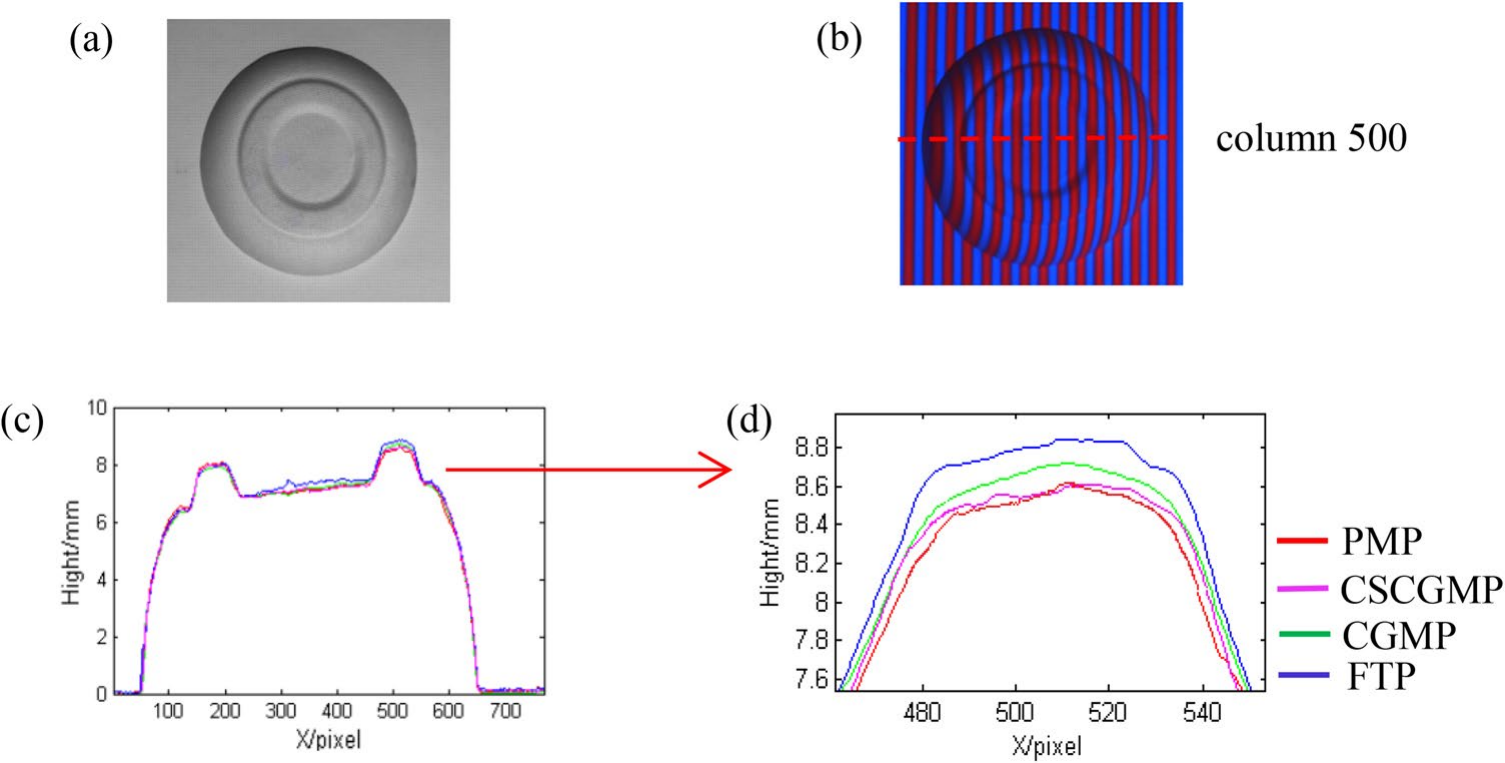
The comparisons of the reconstructed results by the four methods: (**a**) the measured plate; (**b**) the corresponding color deformed pattern of the plate; (**c**) the cutaways at column 500; (**d**) the dashed rectangular box area in (**c**).

In order to verify the accuracy of CSCGMP, a lot of comparative experiments have been done. As is known to all, large-step PMP is the most accurate measuring method in the optical 3D measuring methods based on structure light projection, so a reconstructed object by 8-step PMP^31^ is regarded as quasi truth-value. Here a plate is measured by FTP, CGMP, CSCGMP, and 8-step PMP. The comparisons of reconstruction results are shown in Fig. 9.

Figure 9a shows the measured plate. The corresponding color deformed pattern by CSCGMP is shown in Fig. 9b. The cutaways at column 500 of the reconstructed object by the above four methods are shown in Fig. 9c. For the convenience of detail comparisons, the enlarged figure of the dashed rectangular box area in Fig. 9c is shown in Fig. 9d.

It can be seen from Fig. 9d that the plate reconstructed by CSCGMP is much close to that by 8-step PMP. That means the reconstructed result by CSCGMP is better than that by either FTP or CGMP.

In CGMP, the background light intensity is directly eliminated by filtering the captured deformed pattern. The result of reconstruction may be affected by the spectrum overlapping to some extent. In FTP, the background light intensity of captured deformed patterns is eliminated by filtering operation too. However, in CSCGMP, the background light intensity of the captured color deformed pattern is eliminated by normalizing and subtracting red and blue components of the color deformed pattern. No filtering operation is needed to eliminate the background light intensity by CSCGMP. Thus, the reconstructed object is more accurate by CSCGMP.

Therefore, CSCGMP owns the superiority of single-shot and better accuracy. It has great potential applications in real-time 3D measurement.

## Conclusion

In this paper, a CSCGMP method is proposed. While only one composite color grating in which two sinusoidal gratings with a *π* phase difference are encoded in red and blue channels respectively is projected onto the measured object, the only one corresponding color deformed pattern is needed to be captured. The 3D shape of the measured object can be reconstructed successfully. Since spectrum filtering is avoided effectively in the purification of the AC component of the deformed pattern, it is directly purified in the spatial domain. Even when the spectrums of the DC component and the AC component are overlapped, the purification of the AC component is not affected, so the purification accuracy is higher. Numerical simulation and experimental results verify the effectiveness and practicability of the proposed method. Further comparative experiments show that CSCGMP has higher measurement accuracy than FTP and CGMP, and reserves its single-shot feature, so it has great potential applications in real-time 3D measurement. However, in CSCGMP, because the color grating is used to captured the 3D information of the measured object, measuring color object is still a challenge.

## Supplementary Information

Supplementary Video S1.

Supplementary Video S2.
