# Supplementary figures and images for: Color-encoded single-shot computer-generated Moiré profilometry
Source: Sci Rep. 2021 May 26;11:11020. doi: 10.1038/s41598-021-90522-x (PMC8155059; doi:10.1038/s41598-021-90522-x)

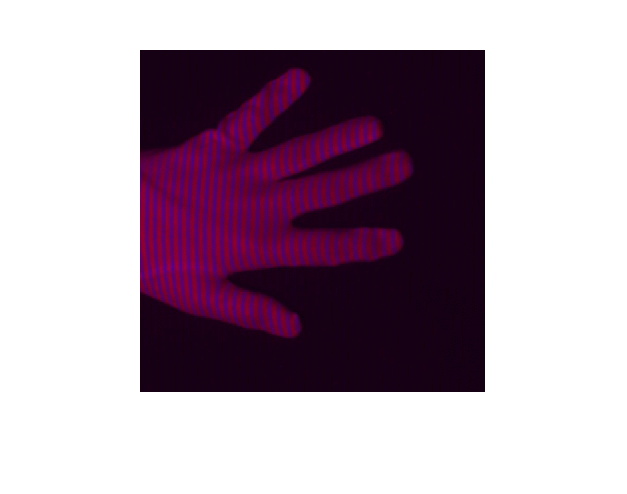

Supplement: Supplementary file 1 — Supplementary Video S1. [file 41598_2021_90522_MOESM1_ESM.gif]

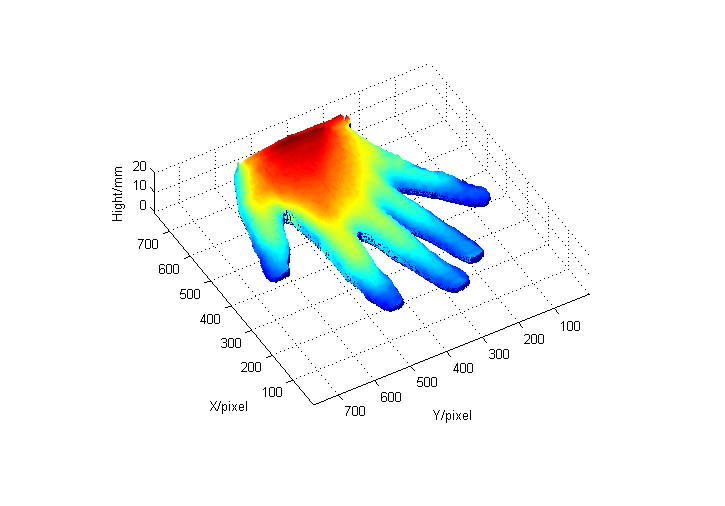

Supplement: Supplementary file 2 — Supplementary Video S2. [file 41598_2021_90522_MOESM2_ESM.gif]
